# National Insurance. The Approved Societies and the Hospitals

**Published:** 1913-11-22

**Authors:** 


### The Hospital

The Workers' Newspaper of Administrative Medicine and Institutional Life, Administration, National Insurance and Health.

No. 1430, Vol. LV. SATURDAY, NOVEMBER 22, 1913. PRICE ONE PENNY.

### NATIONAL INSURANCE, THE APPROVED SOCIETIES, AND THE HOSPITALS.

In The Hospital of November 1, p. 121, we dealt with the effect of the National Insurance Amendment Act on hospital finance. We urged that a complete mutual understanding between the approved societies and the hospitals would be furthered by a full recognition on the part of these societies of the work done by the voluntary hospitals for the good of the insured. The hospitals have the right to look to the approved societies for substantial and adequate financial support in consideration of the benefits conferred upon their sick members, and thus upon the societies as a whole. Unfortunately, the approved societies are not yet, and will not for some time be, in a position to know exactly how they stand in regard to the sufficiency of their funds to meet their liabilities. These societies are therefore at present properly chary of incurring financial responsibilities beyond those they are bound to recognise under the compulsory provisions of the Acts. No doubt this may account, in a measure, for the action of the societies which appear to disregard the claims of the hospitals. It must not be forgotten that in The Hospital of June 14 last, p. 332, a clear statement of the futility of the permission accorded to the approved societies of entering into agreements with the hospitals was convincingly set forth in the report of the London Hospital. Some persons are urging that, in view of the fact that Section 15 of the Amendment Act has now come into operation, the change of opinion which is growing up amongst the larger approved societies and the present altered conditions justify the assumption that where the voluntary hospitals carefully and tactfully make representations to the societies, the result, in many cases, might be an agreement for the treatment of insured persons on a financial basis.

We could wish that these optimistic views were supported by evidence. The approved societies are fully aware that without the hospitals and the aid they are at present rendering by granting free in-patient treatment to insured persons they would be hopelessly insolvent. The greater approved societies have already come to recognise, we are assured, that great advantage would accrue to them if they could rely with certainty on the services of the hospital as in some sense a *quid pro quo* for the contributions made. The supporters of the Government and of the panel system recently put out a feeler, through a correspondent, whose communication appeared in the *Times* of November 1. The main object of this communication appeared to be to obtain help for the panel doctors throughout London from (*a*) the Public Health Services, and (*b*) the voluntary hospitals. This correspondent, like all the present Government's supporters in the matter of insurance, expressed no sense of gratitude or obligation to the hospital authorities for the substantial service they have rendered to insured persons sent to the out-patient department for consultation. But he urged as an ideal arrangement (with commendable discretion he does not say for whom) that the hospital could be the centre for each Insurance area, which every panel doctor should feel that he could use for consultative purposes. He glorified the equipment and organisation of the modern hospital, with all its scientific apparatus for the investigation of disease, and demanded that these expensive equipments should be at the service of the panel doctors, who should be encouraged to take advantage of them, and so add greatly to the expenditure of these institutions. His only reference to payment to the hospitals for these costly and invaluable services is the mention of the recent circular issued by the Insurance Commissioners which we published last week, p. 173, in which "it is made clear that the approved societies may subscribe to hospital funds." He ends by suggesting that a conference should be arranged between representatives of the London hospitals and the London Insurance Committee, with a view to organise facilities for hospital treatment for panel patients, with scientific investigation on a broad and comprehensive basis.

We have here, therefore, two points urged upon the hospitals: (*a*) to seek agreements with the larger approved societies, at any rate, with a view to arrangements being made for the admission of patients by subscriptions on a financial basis; and (*b*) for the London hospitals to confer with the Insurance Committee, with a view to sanctioning a much larger expenditure of hospital funds upon insured persons than prevails at present. We have no doubt, in view of their past experience, that the London hospitals will exhibit commendable caution in this matter, and that their example will be followed by the voluntary hospitals throughout the country. The wording of the October circular of the Insurance Commissioners (A.S. 106) makes it abundantly clear that the approved societies are not in a position at present to set apart funds for the purpose of subscribing to the hospitals. In the case of all but the largest societies great difficulty has been experienced in confining their expenses to the prescribed limits; and in the case of those larger approved societies, where a surplus may be estimated to exist, this surplus, derived by a saving in administrative expenses, has been ear-marked as "a special reserve fund, to guard against a possible deficiency, as the result of adverse experience when the first valuation is made in 1917," or "as a source of profit, out of which additional benefits are to be granted to the insured."

It follows that the Commissioners' circular must be regarded by men of business throughout the hospital world as another instance of make-believe, a policy which the present Government has reduced to a fine art, so far as some aspects of National Insurance are concerned. By all means let every hospital manager throughout the country study the points we have brought out in this article with the utmost care and impartiality. Let them question the Insurance Commissioners closely in view of their October circular, so that they may obtain a statement of the available funds, which in their view the approved societies have at their disposal or discretion, out of which they may contribute substantially towards the cost the voluntary hospitals have been put to by the treatment of insured persons. In this way they will at least be in a position to judge whether the circular is worth more or less than the promises of the Chancellor of the Exchequer, and the prospects of benefit to the hospitals offered in the original Act, which have proved in practice to be valueless. Further, let each hospital get into communication with the approved societies from which their registers show insured persons have been received for treatment. Let them refer to the Commissioners' circular and ask the societies kindly to intimate if they have any funds at all available for the purpose indicated by the Commissioners, and if so, in what sums, and from what date they are prepared to subscribe to the voluntary hospital.

We should be very much indebted to the managers of any hospital, which takes the course here recommended, if they will kindly keep us informed of the results which follow the adoption of this policy. It is perfectly clear that the voluntary hospitals have one asset which they should make the most of—the time between now and the date of the first valuation in 1917. This time should be utilised to the full in making the case of the voluntary hospitals known to the public and to provide by co-operation that the strength of that case shall be brought out clearly and unanswerably when the crisis comes, and a remedy for sweating the hospitals has to be found, as found it must be.

